# Connexins in cancer: bridging the gap to the clinic

**DOI:** 10.1038/s41388-019-0741-6

**Published:** 2019-02-27

**Authors:** Trond Aasen, Edward Leithe, Sheila V. Graham, Petra Kameritsch, María D. Mayán, Marc Mesnil, Kristin Pogoda, Arantxa Tabernero

**Affiliations:** 1 Translational Molecular Pathology, Vall d’Hebron Institute of Research (VHIR), Autonomous University of Barcelona, CIBERONC, Barcelona, Spain; 2 Department of Molecular Oncology, Institute for Cancer Research, Oslo University Hospital and K.G. Jebsen Colorectal Cancer Research Centre, Oslo University Hospital, Oslo, Norway; 3 MRC-University of Glasgow Centre for Virus Research, Institute of Infection, Immunity and Inflammation, College of Medical, Veterinary and Life Sciences, University of Glasgow, Glasgow, UK; 4 Walter Brendel Centre of Experimental Medicine, Ludwig-Maximilians-Universität München and Munich University Hospital, München, Germany; 5 CellCOM Research Group, Instituto de Investigación Biomédica de A Coruña (INIBIC), Servizo Galego de Saúde (SERGAS), University of A Coruña, A Coruña, Spain; 6 STIM Laboratory, Faculté des Sciences Fondamentales et Appliquées, Université de Poitiers, Poitiers, France; 7 Departamento de Bioquímica y Biología Molecular, Instituto de Neurociencias de Castilla y León (INCYL), Universidad de Salamanca, Salamanca, Spain

**Keywords:** Cancer therapy, Diagnostic markers, Cancer microenvironment

## Abstract

Gap junctions comprise arrays of intercellular channels formed by connexin proteins and provide for the direct communication between adjacent cells. This type of intercellular communication permits the coordination of cellular activities and plays key roles in the control of cell growth and differentiation and in the maintenance of tissue homoeostasis. After more than 50 years, deciphering the links among connexins, gap junctions and cancer, researchers are now beginning to translate this knowledge to the clinic. The emergence of new strategies for connexin targeting, combined with an improved understanding of the molecular bases underlying the dysregulation of connexins during cancer development, offers novel opportunities for clinical applications. However, different connexin isoforms have diverse channel-dependent and -independent functions that are tissue and stage specific. This can elicit both pro- and anti-tumorigenic effects that engender significant challenges in the path towards personalised medicine. Here, we review the current understanding of the role of connexins and gap junctions in cancer, with particular focus on the recent progress made in determining their prognostic and therapeutic potential.

## Introduction

**Fig. 1:**
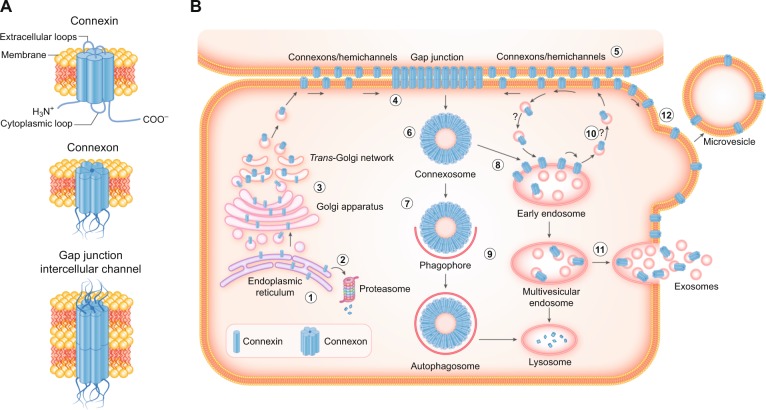
Connexins, connexons and gap junctions. **a** Connexins are tetraspanning integral membrane proteins with cytosolic C and N termini. Six connexins oligomerize to form a connexon. At the plasma membrane, the connexon can dock head-to-head with a connexon in an adjacent cell to form a gap junction intercellular channel. **b** Biosynthesis, intracellular trafficking and degradation of connexins. (1) Connexins are cotranslationally inserted into the endoplasmic reticulum. (2) A subpool of newly synthesised connexins undergo endoplasmic reticulum-associated degradation. (3) During their trafficking to the plasma membrane, connexins oligomerise into connexons. (4) After their arrival at the plasma membrane, the connexons can dock with connexons from adjacent cells to form gap junction channels. (5) Connexons may also form functional channels at the non-junctional areas of the plasma membrane (also known as hemichannels). (6) Gap junction endocytosis results in the formation of a connexosome (also known as annular gap junction). (7) The connexosome may be degraded by autophagy. (8) Alternatively, the connexosome may change its morphology to that of a connexin-enriched multivesicular endosome in a process that is associated with the fusion between the connexosome and early endosomes. (9) Connexins are sorted from early endosomes to lysosomes via late endosomes. (10) Under certain conditions, endocytosed connexons may undergo recycling to the plasma membrane. Hemichannels also undergo endocytosis and recycling, but their endocytic and recycling pathways are poorly characterised (question marks). (11) Multivesicular endosomes can fuse with the plasma membrane to secrete exosomes containing connexons. (12) Microvesicles containing connexons can be formed by the outward budding of the plasma membrane

Connexins are integral membrane proteins that form channels between adjacent cells and thereby permit the bidirectional cytosolic exchange of ions, metabolites and secondary messengers ( < ~ 1200 Da) (Fig. 1a) [1]. These channels assemble into distinct plasma membrane domains termed gap junctions (Fig. 1b) [1]. Gap junction intercellular communication (GJIC) plays essential roles in tissue homoeostasis and regulation of cell growth and differentiation [2]. In addition, connexins form functional channels at the non-junctional areas of the plasma membrane (Fig. 1) [3]. These so-called hemichannels provide a communication pathway between the intracellular and extracellular milieus that is important in autocrine and paracrine signalling [3]. Connexins also possess significant channel-independent roles, including the one as signalling hubs, which may occur at the plasma membrane, in the cytoplasm or even in the nucleus [2].

The human connexin protein family contains 21 members, of which the most widely studied is connexin 43 (Cx43) [2]. A large body of experimental evidence suggests that connexins are causally involved in cancer pathogenesis, possessing both pro- and anti-tumorigenic functions (see the below section and recent reviews [2, 4]). Significant advances have been made towards understanding how connexins can act as either tumour suppressors or tumour promoters depending on the isoform, cancer stage and tissue. In addition to the emerging prognostic value of connexins in cancer, several studies indicate that connexin targeting might have considerable therapeutic implications [5, 6]. This review provides an overview of our current understanding of the role of connexins in cancer, with emphasis on their prognostic and therapeutic potential.

## Complex roles of connexins in cancer

### Connexins as tumour suppressors

**Fig. 2:**
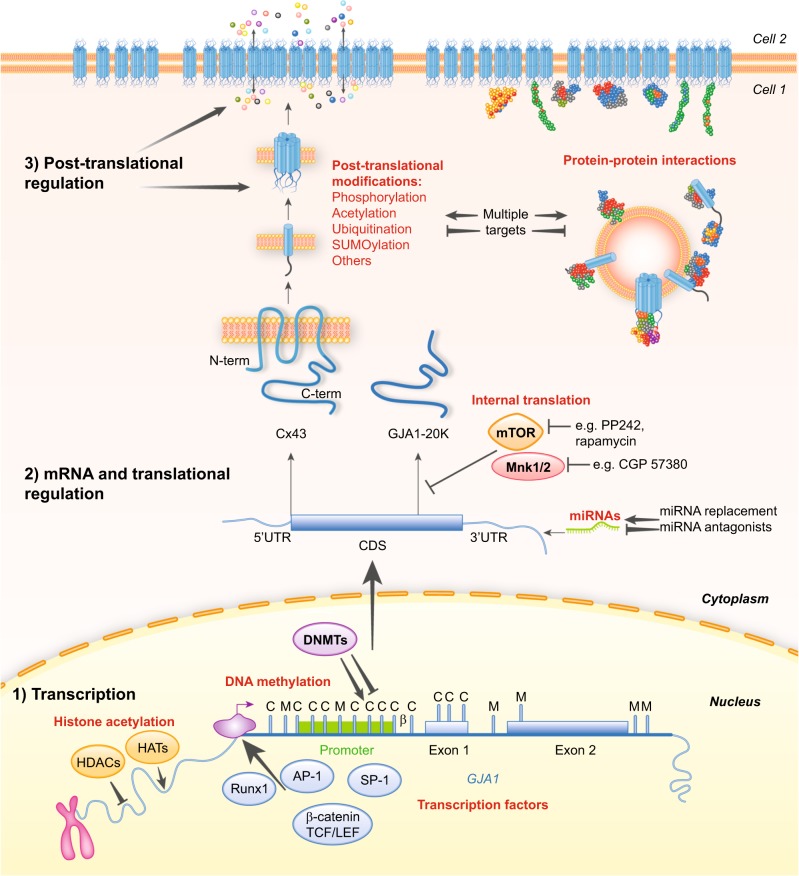
Dysregulation of connexins in cancer: therapeutic opportunities. Multiple stages of connexin life cycle are subject to dysregulation during cancer progression, as exemplified by *GJA1* (Cx43). (1) Transcription: connexin expression is often reduced (but sometimes increased) in human tumours at the mRNA expression level, of which multiple pathways are therapeutic targets (text highlighted in red for key targets), including transcription factor activity and epigenetic silencing by histone acetylation and promoter methylation (promoter region in green, with C and M illustrating the non-methylated and methylated sites, respectively; blue, some important transcription factors regulating Cx43 expression). Histone acetylation can be modified by targeting histone acetyltransferase enzymes (HATs) or histone deacetylases (HDACs), typically promoting and repressing transcription, respectively. Transcriptional silencing due to promoter hypermethylation by DNA methyltransferase enzymes (DNMTs) may also be amenable to therapeutic intervention leading to the restoration of GJIC. (2) mRNA regulation: mRNA stability and translation is subject to regulation by multiple cancer-associated microRNAs. Moreover, alternative translation initiation, resulting in the synthesis of truncated forms of Cx43, might regulate Cx43 and have important implications for its dysregulation in cancer. This process is regulated by key cancer signalling pathways such as mTOR and Mnk1/2 and is altered during pathological conditions such as hypoxia. Truncated forms of Cx43, notably the 20-kDa form named GJA1–20k, may be important for the efficient targeting of Cx43 to the membrane. Indeed, Smad3/ERK-dependent repression of GJA1–20k was recently shown to reduce Cx43 gap junctions during epithelial-to-mesenchymal transition (EMT). (3) Post-translational regulation: connexins frequently display an aberrant localisation in cancer cells. Phosphorylation and other multiple post-translational events, occurring mainly at their C terminus, regulate connexin trafficking and stability at the plasma membrane. Cx43 is regulated by several kinases that are frequently overactivated or overexpressed during cancer development and susceptible to pharmacological inhibition, such as mitogen-activated protein kinase (MAPK), protein kinase C (PKC), protein kinase A (PKA), cdc2/cyclin B and v-src/c-src. Cx43 is also regulated by acetylation, ubiquitination and SUMOylation

The idea that GJIC regulates tumour growth was proposed by Loewenstein and Kanno more than 50 years ago in a seminal ex vivo study, in which they demonstrated the loss of electrical coupling occurring in rat liver tumours [7]. Since then, several connexin knockout mouse models have supported the notion that connexins have tumour-suppressive functions (reviewed in ref. [2]), notably Cx32 (multiple organs), Cx43 (breast and lung) and Cx26 (breast). Cancer cells often display a loss of GJIC due to the dysregulation of connexin expression at multiple levels (Fig. 2). At the transcriptional level, the reduced connexin expression in tumours involves transcriptional and epigenetic mechanisms such as promoter methylation [8–11]. At the post-transcriptional level, the suppression of connexin expression can be due to connexin-targeting microRNAs [12, 13]. At the protein-synthesis level, internal translation of N-terminally truncated forms of Cx43 [14–16] has recently been described to regulate connexin membrane targeting [14], and Smad3/ERK-dependent repression of this process was reported to reduce Cx43 gap junctions during epithelial-to-mesenchymal transition [17]. At the post-translational level, many growth factors, oncogenes and tumour-promoting chemicals are potent inducers of Cx43 phosphorylation, which is often associated with the inhibition of GJIC [18] and increased Cx43 ubiquitination, endocytosis and degradation [19].

In accordance with the notion that connexins might act as tumour suppressors, the ectopic expression of connexins in cancer cells often partly restores growth control (e.g. refs. [20–25]) and differentiation potential (e.g. refs. [26–28], reviewed in ref. [2]). Conversely, the experimental depletion of connexins may result in more aggressive cancer cell growth [29]. In addition to their role in modulating cell proliferation [30], connexins can either promote or prevent cell death by apoptosis [31]. Such effects may be due to the gap junction-mediated intercellular passage of survival or death signals such as Ca^2+^, IP_3_ and cAMP [2, 32–34]. Moreover, hemichannels may exchange proapoptotic and survival factors between extracellular and intracellular environments [35].

**Fig. 3:**
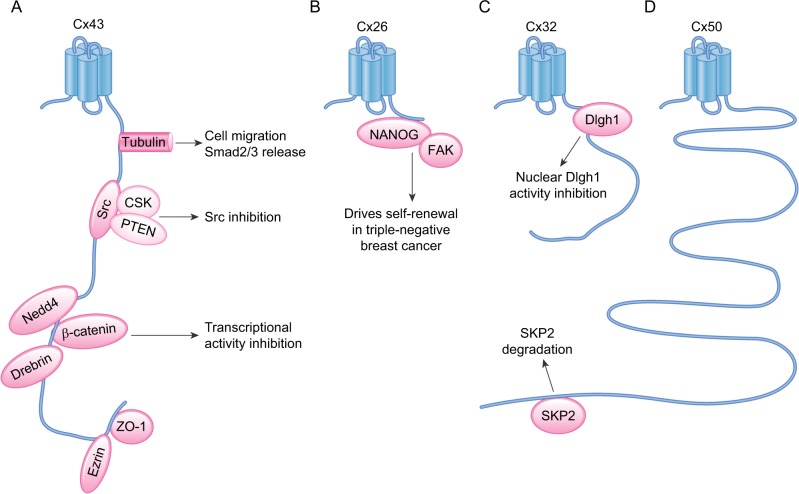
Interactions between connexins and proteins that affect tumour growth and migration. Examples of proteins that interact with specific regions of connexins and may act as therapy targets. **a** The interaction between Cx43 and tubulin is involved in the regulation of cell migration. Similar mechanisms have been proposed for other proteins associated with the cytoskeleton, such as cadherins, catenins, vinculin, ZO-1 and drebrin. In addition, Cx43 may compete with the tubulin–Smad2/3 interaction causing Smad2/3 release. Cx43 binds to c-Src and its endogenous inhibitors CSK and PTEN, promoting c-Src inhibition. Cx43, by interacting with β-catenin, prevents the transcriptional activity of β-catenin in the nucleus, where it regulates the expression of genes involved in promoting cell malignancy. A similar sequestration mechanism may occur with drebrin, ezrin or ZO-1. These proteins, and many others such as Nedd4, also have important roles in regulating Cx43 gap junction plaques, which influence GJIC and therefore may have therapeutic potential. **b** Cx26 has been proposed to maintain a cancer stem cell phenotype specifically in triple-negative breast cancer cells through its interaction with NANOG and focal adhesion kinase (FAK). **c** Cx32 binds to the scaffold protein discs large homologue 1 (Dlgh1) and may control cell proliferation in liver cells through its interaction with and maintenance of Dlgh1 at the plasma membrane. The interaction of Dlgh with Cx43 has also been associated with cancer progression through a mechanism involving the oncoprotein E6 (see section “Connexins and tumour viruses”). **d** Cx50 interacts with and promotes auto-ubiquitination and the subsequent degradation of Skp2, a key negative regulator of the cyclin-dependent kinase (CDK) inhibitor p27. **a**–**d** To complicate this scenario, the phosphorylation of connexins modifies their binding affinities to various protein partners. For instance, c-Src phosphorylation affects the binding of several Cx43 partners. GJIC, gap junction intercellular communication

There is increasing evidence that connexins can suppress the growth of cancer cells through channel-independent mechanisms [22, 30, 36–39] (Fig. 3). For example, the ectopic expression of the intracellular C terminus (CT) of Cx43 can in some cases inhibit cell proliferation to a similar extent as full-length protein [24]. Connexins may also modulate the activity of some of their partners by affecting their cellular location, as proposed by Skp2 for Cx50 [40], β-catenin for Cx43 [38], discs large homologue 1 (Dlgh1) for Cx32 [41] and Cx43 [42], or by other mechanisms, such as the recruitment of Src together with its endogenous inhibitors CSK and PTEN resulting in a switch from the active to inactive conformation of c-Src [43] (Fig. 3). Because connexins present a low level of homology within their CT sequences, the channel-independent regulation of cell growth is expected to vary considerably among different isoforms.

### Connexins as promoters of invasion and metastasis

When assessing the role of connexins in cancer pathogenesis, we must consider that their ability to act as tumour suppressors can vary considerably among tissue types and cancer stages, as well as among connexin isoforms [2]. Furthermore, there is growing evidence that some connexin isoforms are protumorigenic under certain conditions. For example, connexins can promote the migration and invasion of tumour cells [44, 45]. In addition, connexins can form heterologous gap junctions between tumour cells and endothelial cells to facilitate intravasation and extravasation [46–49]. Connexins can also nurture metastatic growth and may promote resistance to cancer treatments [50–53]. In accordance with this notion, a recent study demonstrated that brain metastatic cancer cells establish gap junctions with astrocytes to promote tumour growth and chemoresistance [50].

### Connexins and cancer stem cells

Emerging experimental evidence indicates that connexins play important roles in cancer stem cell (CSC) biology. Malignant glioma stem cells (GSCs) express very low levels of Cx43, and GSC stemness is reduced by its transfection with Cx43 or treatment with peptides containing sequences of Cx43 that interact with c-Src (Cx43 mimetic peptides) [54, 55]. A recent work has found that the expression levels of Cx43 and Cx46 were increased and reduced, respectively, during GSC differentiation and that targeting of Cx46 compromises GSC maintenance [56]. While Cx43 is almost absent in liver CSCs [57], the accumulation of cytoplasmic Cx32 has been associated with metastasis and enhanced self-renewal of CSCs in human HuH7 hepatoma cells [58]. Some subtypes of breast cancer cells positive for the putative CSC marker CD44 express Cx43 [59, 60]. Moreover, Cx43 clearly plays a subtype-dependent role in breast cancer [61]. In triple-negative breast cancers, Cx26 was shown to be upregulated in CSC population, in which it forms a complex with NANOG and focal adhesion kinase to drive self-renewal (Fig. 3) [62].

Overall, connexins can be both anti- and pro-tumorigenic, acting via processes that include the regulation of CSCs, and this depends on the connexin isoform and tissue type. Understanding this complexity is the key to the development of new and efficient therapies.

## Prognostic value of connexins in cancer

A number of studies suggest that connexins can be independent tumour markers predicting both better and worse prognoses. Further insights into this seeming contradiction have been provided by recent analytical tools for public databases. The Pathology Atlas within the Human Protein Atlas database (https://www.proteinatlas.org/pathology) includes an open-access database (retrieved from The Cancer Genome Atlas (TCGA)) containing correlation analyses based on mRNA expression levels with respect to the clinical outcome for 17 major cancer types and almost 8000 cancer patients [63]. Those mRNAs encoding connexins identified to have a highly significant prognostic value (*P* < 0.001) in any of these cancer types are summarised in Table 1. Notably, *GJA1* (encoding Cx43) is featured in the list of 20 genes most significantly associated with an unfavourable prognosis in stomach cancer. Indeed, for most connexins and cancer types, high levels of connexin mRNA expression are associated with a poor prognosis. It is also notable that the mRNA expression of a given connexin-encoding gene can be both favourable and unfavourable, depending on the type of tumour (Table 1). Different connexins can also have differing prognostic prediction results for the same type of tumour (e.g. Cx26 vs Cx32 in renal cancer). The sub-classification of cancers is also relevant. For example, the Kaplan–Meier Plotter tool (http://kmplot.com/analysis) suggests that high levels of Cx43 mRNA are associated with good and poor prognoses in oestrogen receptor-positive and -negative breast cancer subtypes, respectively [64].

**Table 1:** Significant associations between connexin mRNA expression and cancer prognosis

Connexin (gene)	Cancer	Clinical relevance	Survival,^a^ % alive	*P* value (best separation)
Cx26 (*GJB2*)	Renal	U	*H* = 52% (*n* = 188), *L* = 73% (*n* = 689)	5.98E-04
	KIRC	U	*H* = 46% (*n* = 130), *L* = 70% (*n* = 398)	7.79E-05
	Pancreatic	U	*H* = 15% (*n* = 86), *L* = 40% (*n* = 90)	2.90E-04
	Glioma	U	*H* = 0% (*n* = 39), *L* = 11% (*n* = 114)	5.98E-04
	Lung^b^	U	*H* = 43% (*n* = 795), *L* = 49% (*n* = 199)	6.25E-04
	LUAD^b^	U	*H* = 33% (*n* = 330), *L* = 53% (*n* = 170)	1.45E-05
Cx30.3 (*GJB4*)	Pancreatic	U	*H* = 15% (*n* = 139), *L* = 66% (*n* = 37)	3.24E-05
	Cervical	F	*H* = 74% (*n* = 181), *L* = 52% (*n* = 110)	5.48E-04
Cx31 (*GJB3*)	Pancreatic	U	*H* = 15% (*n* = 70), *L* = 37% (*n* = 106)	7.56E-05
	Lung^b^	U	*H* = 44% (*n* = 709), *L* = 45% (*n* = 285)	4.56E-04
	LUAD^b^	U	*H* = 18% (*n* = 107), *L* = 45% (*n* = 393)	3.54E-08
Cx31.1 (*GJB5*)	Pancreatic	U	*H* = 0% (*n* = 66), *L* = 42% (*n* = 110)	1.54E-04
Cx32 (*GJB1*)	Renal^b^	F	*H* = 77% (*n* = 451), *L* = 61% (*n* = 426)	2.85E-06
	KIRC^b^	F	*H* = 75% (*n* = 284), *L* = 50% (*n* = 244)	4.88E-08
Cx37 (*GJA4*)	Renal^b^	U	*H* = 67% (*n* = 700), *L* = 80% (*n* = 177)	7.44E-04
	KIRC^b^	F	*H* = 68% (*n* = 417), *L* = 48% (*n* = 111)	8.57E-04
	KIRP^b^	U	*H* = 58% (*n* = 77), *L* = 82% (*n* = 208)	1.07E-05
	Liver	F	*H* = 53% (*n* = 225), *L* = 39% (*n* = 140)	3.28E-04
Cx43 (*GJA1*)	Stomach	U	*H* = 14% (*n* = 98), *L* = 45% (256)	4.99E-05
	KIRC^b^	F	*H* = 68% (*n* = 409), *L* = 45% (119)	2.60E-04
Cx45 (*GJC1*)	Renal	U	*H* = 63% (*n* = 562), *L* = 80% (*n* = 316)	3.55E-08
	Urothelial	U	*H* = 34% (*n* = 286), *L* = 58% (*n* = 120)	7.83E-04

^a^Survival: percentage of patients alive after 3 years for glioma and 5 years for all other cancer types. The table and the *P* values are based on a best-fit model in which patient numbers (*n*) are stratified into high (H) and low (L) connexin expression groups

^b^Cancer subtype-specific evidence (i.e. only some subtypes correlate). All data are taken from the Human Protein Atlas (https://www.proteinatlas.org/pathology) after analysis by Uhlen et al. [63]. Fragments per kilobase million (FPKM) cut-off (median separation) is set to 1

*F* connexin expression is favourable for outcome, *U* connexin expression is unfavourable for outcome, *H* high expression, *L* low expression, *n* number of patients, *LUAD* lung adenocarcinoma, *KIRC* kidney renal clear cell carcinoma, *KIRP* kidney renal papillary cell carcinoma

Several specific studies have also reported a correlation between connexin mRNA levels and survival or phenotypic features. For instance, high levels of Cx43 mRNA in glioma tumours have been correlated with poor survival [65]. Expression of connexin mRNA in the surrounding tissue may also correlate with a specific tumour phenotype or behaviour. For example, in melanoma, the increased expression of Cx26 and Cx30 at the mRNA level in the surrounding skin keratinocytes is significantly correlated with malignant features such as tumour thickness and, in the case of Cx26, metastasis [66]. In lung cancer, the reduced expression of Cx43 mRNA in adjacent normal lung tissue (due to promoter methylation) is significantly correlated with nodal involvement, suggesting that Cx43 could be a marker for micrometastasis in non-small cell lung cancer [8]. Recently, Cx30.3 (*GJB4*) mRNA expression was shown to be increased in lung tumours, with the levels in blood buffy coat samples serving as a biomarker for diagnosis and poor prognosis [67]. Mechanistically, Cx30.3 was shown to promote tumour growth and induce chemoresistance via the MET-induced activation of Src.

As shown in Table 1, Cx26 mRNA expression is associated with a poor prognosis in various cancers. For example, high levels of Cx26 mRNA are associated with reduced overall survival in pancreatic cancer (*P* < 0.001) [68]. Microarray analysis (and confirmatory quantitative real-time polymerase chain reaction analysis) of breast cancer samples show the upregulation of Cx26 during progression from ductal carcinoma in situ to invasive ductal carcinoma [69]. At the protein level, Cx26 is detected in the invasive carcinoma foci [69]. In agreement with these findings, Cx26 was recently found to drive CSC self-renewal in triple-negative breast cancer [62].

At the DNA level, few studies have provided an insight into the role of connexins in cancer. Mutations in Cx43 have been described in advanced metastatic colon cancer lesions [70]. However, current large-scale genetic studies and analytical tools (e.g. Intogen https://www.intogen.org or COSMIC http://cancer.sanger.ac.uk/cosmic) suggest relatively low mutation rates in connexin-encoding genes and, based on genetic data, none of the 21 connexin genes are classified as tumour suppressors or oncogenes. Nevertheless, individual genetic studies merit follow-up. For example, the C1019T polymorphism in *GJA4* (encoding Cx37) is associated with *Helicobacter pylori* infection in patients with gastric cancer [71]. In glioma, *GJB6* (encoding Cx30) was deleted in 25.8% of the 751 analysed tumours and was mutated in 15.8% of 158 tumours [51]. However, again, the overall patient survival was not correlated with the presence of *GJB6* deletions (517 tumours from TCGA and 67 tumours from REMBRANDT) or *GJB6* mRNA expression (in patients from TCGA). However, at the protein level, high Cx30 expression adversely influenced the survival (Table 2).

**Table 2:** Direct correlations between connexin protein levels and patient survival

Cancer	Cx	Association	Clinical relevance	*P* value	Comments	Reference
Bladder	Cx43	U	High Cx43 = reduced PFS and RFS in stage pTa and pT1 patients	*P* < 0.001	High Cx43 associated with a higher tumour grade, multiplicity and increased proliferation (all *P* < 0.05)	Poyet et al. [190]
Bone	Cx43	F	Reduced Cx43 = reduced PFS	*P* = 0.024	Study examined giant-cell tumour of bone	Balla et al. [191]
Breast	Cx26	U	High Cx26 = worse prognosis	*P* < 0.001	Cx26 was correlated with a large tumour size (*P* = 0.013) and a high histological grade (*P* = 0.043). Multivariate analysis: Cx26 (*P* < 0.05) expression an independent prognostic factor	Naoi et al. [192]
	Cx26	U	Decreased Cx26 expression ( < 5%) post-chemotherapy = better OS	*P* = 0.011	Connexin expression may improve the assessment of the pathological response and refine intermediate prognostic subgroups after neoadjuvant chemotherapy	Teleki et al. [193]
	Cx46	F	Cx46 expression ( > 20%) pre- and post-chemotherapy = better OS in the intermediate prognostic subgroups	*P* = 0.002–0.05		
	Cx26	F	High Cx26 = better RFS	*P* = 0.013	Contradicted earlier study findings at the mRNA level (see ref. [193])	Teleki et al. [64]
	Cx32	U	High Cx32 = worse RFS	*P* = 0.027	High Cx32 mRNA expression = improved RFS. Antibody staining not shown	
	Cx30	U	High Cx30 = reduced RFS in grade 3 patients	*P* = 0.016	Independent prognostic markers, but mRNA analysis suggested high tumour subtype-specific differences	
	Cx43	F	High Cx43 = better RFS	*P* = 0.026		
	Cx43	F	High Cx43 = better OS	*P* < 0.001	Independent predictor of survival and distant metastasis-free survival	Chasampalioti et al. [194]
Colorectal	Cx26	U	High Cx26 expression (cytoplasm) = shorter DFS Shorter DFS (*P* = 0.041) and lung metastasis-free survival (*P* = 0.028)	*P* = 0.041 *P* = 0.028	High expression = less-differentiated histology (*P* = 0.0053) and venous invasion (*P* = 0.0084). Lung metastases, but not liver and lymph node metastases, expressed higher Cx26 than the CRC series or corresponding primary CRCs (*P* < 0.0001 and *P* = 0.0001, respectively)	Ezumi et al. [77]
	Cx26	F	Cx26-positive tumours associated with significantly longer survival than Cx26-negative tumours	*P* = 0.0128	Cx26 = independent prognostic factor (*P* < 0.05). Correlation identified between Cx26 and recurrence, histology and p53 expression (*P* < 0.05)	Nomura et al. [195]
	Cx26	U	High Cx26 = shorter patient survival	*P* = 0.02	Cx26 associated with a higher tumour grade (*P* = 0.02)	Knosel et al. [196]
	Cx43	F	Loss of Cx43 expression = shorter relapse-free survival and OS	*P* = 0.017	Depends on subcellular Cx43 staining. Not significant if more advanced stage III and IV tumours are analysed	Sirnes et al. [38]
Oesophageal SCC	Cx26	U	High levels = reduced OS	*P* = 0.007	Correlated with lymph node metastasis (*P* = 0.014) and the number of metastatic lymph nodes (*P* = 0.047)	Inose et al. [197]
	Cx43	U	High levels = reduced OS	*P* = 0.018	Independent prognostic indicator	Tanaka et al. [198]
Gastric	Cx26	F	High Cx26 = better OS	*P* = 0.002	Negative associations between Cx26 expression and clinicopathologic features (all *P* < 0.05). High Cx26 associated with histological intestinal-type (*P* = 0.017) and early-stage gastric carcinoma. Positive Cx26 expression an independent prognostic predictor of intestinal-type GC (*P* = 0.023, hazard ratio = 2.019)	Liu et al. [199]
Glioma	Cx30	U	High Cx30 = worse OS	*P* < 0.05	Correlated with patient survival after irradiation. No correlation at DNA or mRNA level	Artesi et al. [51]
HNSCC	Cx43	F	Cx43 expression = better disease-specific survival of patients	*P* = 0.004	Cx43 positively correlated with p53 expression (*P* = 0.036). No statistical association between Cx43 levels and T, N or M stage	Danos et al. [200]
Liver (hepatitis B-related) HCC	Cx43	F	High Cx43 = delayed tumour recurrence (*P* = 0.001), longer DFS (*P* = 0.026) and OS (*P* = 0.000) in patients with serum alpha-fetoprotein < 400 ng/ml	All *P* ⩽ 0.026	In patients with serum alpha-fetoprotein < 400 ng/ml, Cx43 expression is an independent predictor of later recurrence and longer OS	Wang et al. [201]
Lung	Cx26	U	Cx26-positive SCC tumours associated with increased survival (cancer-related deaths, not OS)	*P* = 0.0391	Multivariate analysis demonstrated that Cx26 expression (*P* = 0.0448) is an independent prognostic predictor	Ito et al. [202]
	Cx43	F	Better mean OS and PFS in NSCLC patients with high Cx43, including the chemotherapy-responder group	*P* < 0.001	Cx43, a reliable surrogate marker for predicting the chemotherapy response and prognosis of patients with advanced NSCLC	Du et al. [203]
Oral SCC	Cx43	U	High membrane expression of Cx43 = short OS	*P* = 0.0088	No correlation for Cx26 or Cx45. Cx43 expression in dysplasia-free mucosa may indicate a very early stage of tumour progression	Brockmeyer et al. [204]
Pancreatic	Cx43	F	Reduced Cx43 associated with higher TNM stage and lymph-node metastasis	*P* < 0.001	Also associated with the degree of differentiation (*P* = 0.002)	Liang et al. [205]
Prostate	Cx26	F	Low Cx26 expression in noncancerous tissue in prostatectomy sections = reduced BRFS and risk of metastasis	*P* = 0.002	Cx26 is only predictive when assessed in the noncancerous areas of the tissue	Bijnsdorp et al. [206]
	Cx43	F	Reduced Cx43 = shorter postoperative BRFS	*P* < 0.001	Patients with lower Cx43 have higher preoperative PSA levels	Benko et al. [207]
	Cx43	F	Reduced Cx43 = shorter postoperative BRFS	*P* < 0.001	Reduced levels of Cx43 associated with high levels of preoperative PSA, a high Gleason score, an advanced pT stage and seminal vesicle invasion (all *P* < 0.05)	Xu et al. [208]
Sarcoma (EWS/PNET)	Cx43	U	Cx43 score correlated with OS	*P* = 0.025	Possible oncogenic and prognostic roles for Cx43 and Cx26 in EWS/PNET. The lack of membranous staining suggests a GJIC-independent mechanism	Bui et al. [209]

*F* connexin expression is favourable for outcome, *U* connexin expression is unfavourable for outcome, *BRFS* biochemical recurrence-free survival, *CRC* colorectal cancer, *DFS* disease-free survival, *EWS/PNET* Ewing’s sarcoma/primitive neuroectodermal tumour, *GC* gastric cancer, *GJIC* gap junctional intercellular communication, *OS* overall survival, *HCC* hepatocellular carcinoma, *HNSCC* head and neck squamous cell carcinoma, *PFS* progression-free survival, *PSA* prostate-specific antigen, *RFS* relapse-free survival, *NSCLC* non-small cell lung cancer, *SCC* squamous cell carcinoma, *TNM* tumour node and metastases

A number of immunohistochemical studies of connexins in cancer—most on Cx26 and Cx43—have described specific prognostic associations. As with mRNA-expression studies, connexin protein expression has been associated with both good and poor prognoses. The studies specifically reporting the significant findings related to patient survival are summarised in Table 2.

In accordance with mRNA-based studies, the expression of different connexins at the protein level can have an opposing prognostic value depending on cancer type, cancer subtype and connexin isoform. For example, in the same breast cancer series, elevated Cx43 and Cx30 levels have been associated with improved and worse breast cancer outcomes, respectively [64]. Another shared finding between the mRNA and protein studies is the observation that high levels of Cx26 are associated with a poor prognosis in most studies/cancers (Tables 1 and 2). This observation is supported by additional studies evaluating tumour histology or aggressiveness, but not the overall survival, including those of thyroid cancer [72] and bladder cancer [73]. Connexin expression has also been proposed as a diagnostic aid in relation to specific tumour subtypes. In gastric cancer, Cx30 was proposed as a potential marker for an intestinal-type cancer phenotype, and the negative expression of Cx30 correlates with a more advanced T grade ( < 0.0001), N grade (0.0123) and tumour stage (0.0014) [74]. An association between the expression level of connexins and metastasis can be found for several cancer types. For instance, Cx43 expression is reduced in primary gastric cancer but increased in matched metastatic lymph nodes [75]. It should also be noted that, in a number of studies, connexin proteins were overexpressed but mislocalised in the cytoplasm. Prominent examples include Cx26 in pancreatic [76] and colon [77] cancer, and Cx43 and Cx32 in prostate cancer [78]. Nuclear Cx43 has also been reported in a number of tumours (e.g. colon cancer [38] and gliomas [79]). It remains to be seen how this mislocalisation correlates with a recent report showing that nuclear Cx43 (or the CT alone) can act as a direct nuclear transcription factor that induces the expression of N-cadherin, an important regulator of processes including epithelial-to-mesenchymal transition and cell migration [80].

In summary, connexins are potential prognostic markers in cancer, but a number of limitations need to be addressed before any clinical application. The fact that connexins have both pro- and antitumourigenic properties and are associated with both good and poor prognoses, depending on cancer stage and type/subtype as well as connexin isoform, complicates their clinical applicability. Standardisation is also difficult in terms of reliable antibodies and evaluation parameters such as the quantification of subcellular connexin localisation (e.g. membrane vs cytoplasm), which can significantly influence the prediction of the clinical outcome.

## Targeting of connexins in cancer: therapeutic opportunities and challenges

The complex and multifunctional role of connexins in cancer provides a wide spectrum of therapeutic opportunities and challenges. Increasing numbers of drugs, peptides and RNAi approaches are available to inhibit or enhance GJIC, hemichannel activity or connexin protein-signalling activity. Below, we highlight a selection of in vivo studies demonstrating that the cancer phenotype is altered as a direct consequence of such an experimental targeting of connexins.

### Modulation of connexin expression and GJIC

The restoration of GJIC and/or connexin expression has long been a potential therapeutic strategy. GJIC can be augmented either by enhancing the permeability of existing gap junctions or by increasing the expression of connexins and thereby elevating the number of open gap junction channels [81]. Many fungal and plant-based compounds, as well as an increasing number of synthetic chemical compounds, either increase GJIC or prevent the loss of GJIC in response to cellular exposure to tumour promoters, which is often associated with reduced tumour growth in vivo. Compounds shown to modify connexins or GJIC and affect the tumour phenotype in vivo are summarised in Table 3. It is worth noting that many chemotherapeutic agents also increase connexin expression and/or GJIC [82]. For example, docetaxel increases Cx43 expression in murine salivary gland carcinoma to reduce tumour growth [83]. This has implications for combinatorial effects in cancer therapy.

**Table 3:** Modulators of connexins and/or GJIC that alter tumour response in vivo

Compound	Derived from	Mechanism of action/effect	Model/cells used	In vivo tumour effect	Cx	Reference
***GJIC enhancers***						
Resveratrol	Natural phenol produced by multiple plants	Increased Cx43 expression and reduced MAPK and NF-κB activation	Male C57BL/6 mice inoculated with B16F10 cells	Decreased tumour size and prolonged survival time	F	Buhrmann et al. [210]
			Orthotopic implantation of MIA PaCa-2 cells in athymic nu/nu nude mice	Tumour growth inhibition, synergy with gemcitabine in MIA PaCa-2 cells	F	Harikumar et al. [211]
Docetaxel	Chemotherapeutic drug (a semisynthetic analogue of paclitaxel)	Increased Cx43 expression inhibits an IL-1α-induced reduction in GJIC	PC-3 and LNCaP cells injected into male BALB/c nu/nu mice	Decreased tumour size	F	Fukushima et al. [212]
PQ1, PQ7	Quinolones (heterocyclic aromatic organic compounds)	Increased Cx43 expression and GJIC (PQ7 also shown to downregulate Cx46)	PyVT spontaneous mammary tumour mouse model	Significantly reduced tumour growth	F	Shishido et al. [213]
			T47D breast cancer cells subcutaneously injected into the inguinal region of the mammary fat pad	Reduced tumour growth in vivo alone and in combination with cisplatin	F	Shishido et al. [214]
All-trans retinoic acid (ATRA)	An active metabolite of vitamin A	Increased connexin expression	Daoy medulloblastoma cells, implementation in mice	Decreased tumour size	F	Li et al. [215]
			Injection of HepG2 human hepatoma cells into athymic BALB/c nu/nu mice. Injection of ATRA	Increased apoptosis and connexin expression in tumours	F	Wu et al. [216]
Valproic acid	A branched short-chain fatty acid derivative of valeric acid	Increased Cx26 and Cx43 expression	Intracranial implantation of human glioma U87 cells into the brain of nude mice	Enhanced antitumour effect of mesenchymal stem cell-mediated HSV-TK gene therapy in intracranial glioma	F	Ryu et al. [217]
Apigenin, lovastatin	Apigenin: a flavonoid; lovastatin: an HMG-CoA reductase inhibitor	Increased Cx43 expression and GJIC	Injection of MCA38 (mouse colon adenocarcinoma) into C57BL/6 mice	Decreased tumour growth due to a combination of HSV-TK/GCV and apigenin/lovastatin	F	Touraine et al. [218]
Shikonin, notoginsenoside R1, aconitine	Natural compounds	In combination, restored GJIC upon urethane exposure	Urethane-induced lung adenocarcinoma in mice	Decreased incidence of mouse lung carcinogenesis and decreased tumour volume	F	Liu et al. [219]
***GJIC inhibitors***						
Oleamide	Naturally occurring amide derived from the fatty acid oleic acid	Structural inhibition of gap junction channels and decreased GJIC	MDA-MB-231 breast cancer cells	Antimetastatic activity	U	Zibara et al. [85]
			BL6 melanoma cells	Reduced spontaneous metastasis	U	Ito et al. [220]
18‑α‑Glycyrrhetinic acid (18‑α‑GA)	A bioactive triterpenoid found in liquorice	Altered connexon particle packing in gap junction plaques and decreased GJIC	Cx26- and Cx43-mediated heterotypic ‘bystander’ effect between endothelial cells expressing HSV-TK suicide gene and tumour cells	Reduced antitumour effect of mesenchymal stem cell-mediated HSV-TK gene therapy in intracranial glioma	F	Ryu et al. [217]
MI-18, MI-22	Derived from oleamide	Decreased GJIC, structurally inhibited Cx26 and Cx32 channels but not Cx40, Cx43 and Cx45 (possibly also interferes with cytoplasmic connexin functions)	BL6 melanoma cells	Reduced spontaneous metastasis	U	Ito et al., Ohba et al. [220, 221]
Camellia oil	Natural oil rich in oleamide-like oleic acid	Inhibited Cx26 channels (probably structural inhibition) and decreased GJIC	BL6 melanoma cells	Reduced spontaneous metastasis	U	Miura et al. [222]
Meclofenamate	A fenamate (anthranilic acid)-derived, FDA-approved drug for joint and muscular pain, arthritis and dysmenorrhoea	Decreased GJIC (possibly due to steric hindrance)	Breast and lung carcinoma cell lines	Reduced brain metastasis growth, enhanced effect of cytotoxic drugs	U	Chen et al. [50]
Tonabersat	Tonabersat (SB-220453), a novel benzopyran derivative used against migraine in human trials	Decreased GJIC (possibly due to steric hindrance)	Breast and lung carcinoma cell lines	Reduced brain metastasis growth, enhanced effect of cytotoxic drugs	U	Chen et al. [50]
Mefloquine	Licensed anti-malaria drug	GJIC and hemichannel inhibitor (likely structural inhibition)	MCF-7 human luminal breast cancer cells	Inhibition of bone metastasis by limiting calcium signalling	U	Wang et al. [86]
Arsenic trioxide	FDA-approved drug for leukaemia	Downregulation of Cx43 (*GJA1*) gene expression (in co-culture systems)	MCF-7 human luminal breast cancer cells	Inhibition of bone metastasis by limiting calcium signalling	U	Wang et al. [86]
Carbenoxolone^a^	A semisynthetic derivative of glycyrrhetinic acid used in human trials	GJIC inhibition and reduced Cx43 expression	MCF-7 human luminal breast cancer cells	Inhibition of bone metastasis by limiting calcium signalling	U	Wang et al. [86]
			Intracranial glioma model using primary human glioma isolates	Enhanced effect of TRAIL therapy	U	Yulyana et al. [136]
			Breast cancer and melanoma cells using zebrafish and chicken embryo models of brain metastasis	Inhibited brain colonisation by blockade of tumour cell extravasation and blood vessel co-option	U	Stoletov et al. [158]
Gap40	Synthetic peptide	Fewer Cx40 channels in endothelial cells causing decreased GJIC	Subcutaneous injection of B16F10 mouse melanoma cells or TC-1 cells (human papillomavirus oncogene-expressing cells derived from C57/Bl6 lung epithelium)	Reduced tumour angiogenesis and tumour growth	U	Alonso et al. [87]
αCT1	Synthetic peptide	Inhibited Cx43-CT interaction with ZO-1 (and other proteins?). Inhibited Cx43 hemichannels (enhanced GJIC?)	Subcutaneous injection of glioma cells (LN229/GSC) in mice	Sensitised chemoresistant glioblastoma cells to temozolomide	U	Murphy et al. [65]

^a^Potent pannexin channel inhibitor

*F* GJIC or connexin expression is favourable for outcome, *GJIC* gap junction intercellular communication, *HSV-TK* herpes simplex virus thymidine kinase, *TRAIL* TNF-related apoptosis-inducing ligand, *U* GJIC or connexin expression is unfavourable for outcome

However, because connexins under specific conditions can facilitate malignant features, in particular, metastasis or growth of metastases [2], there are situations in which the blockade of GJIC or other functions of connexins may offer distinct therapeutic opportunities. The inhibition of GJIC can be achieved through a number of different mechanisms (Table 3 and recent comprehensive reviews [5, 84]). Despite their non-specificity, many chemical gap junction channel inhibitors have been extensively used, even in vivo. For instance, the GJIC blocker oleamide (a fatty-acid derivative) reduces the formation of pulmonary and hepatic metastatic foci and increases the overall survival of mice injected intravenously with MDA-MB-231 breast cancer cells [85]. Mechanistically, oleamide-induced loss of heterologous GJIC between tumour cells and endothelial cells was suggested to interfere with cancer-cell extravasation. Using a brain metastasis mouse model, Massagué and colleagues recently demonstrated the potent growth inhibition of brain metastases from breast and lung cancer cells in response to blockade of heterologous GJIC between the cancer cells and astrocytes [50]. Inhibition of heterologous GJIC was achieved either by the knockdown of Cx43 expression or by the use of the gap junction channel inhibitors tonabersat or meclofenamate, which pass the blood–brain barrier [50]. Tonabersat has been used as a migraine prophylaxis drug, whereas meclofenamate is an FDA-approved anti-inflammatory drug for oral administration. Mechanistically, these GJIC inhibitors seem to inhibit tumour growth by blocking a cGAMP-mediated signalling cascade that orchestrates paracrine signalling between host and tumour cells [50]. Consequently, as the first cancer clinical trial in which GJIC is specifically being targeted, meclofenamate is being tested in human patients with recurrent or progressive brain metastasis (https://clinicaltrials.gov/ct2/show/NCT02429570?id=NCT02429570&rank=1&load=cart). Recently, a novel role of Cx43-based gap junctions was also identified in breast cancer bone metastasis [86]. Gap junctions were found to mediate calcium flow from osteogenic cells to the cancer cells. Blocking such heterocellular intercellular calcium transfers from the osteogenic niche to cancer cells, by gap junction inhibitors (carbenoxelone and arsenite trioxide) or by the ablation of Cx43 in either the osteogenic cells or the cancer cells, prevented bone metastasis progression in mice [86].

Thus, many of the studies reporting the inhibitory effects on tumour growth upon inhibition of GJIC mechanistically relate this effect to a loss of heterologous GJIC between tumour cells and cells in their microenvironment (as reviewed in [50, 85] and discussed elsewhere in this manuscript). Other successful strategies include the peptide-mediated inactivation of Cx40 in endothelial cells causing the inhibition of tumour angiogenesis in vivo and subsequently decreased tumour growth [87] (see Table 3 and section “Emerging concepts”). αCT1, a peptide that blocks ZO-1 and potentially other proteins from interacting with the Cx43-CT (see section “Targeting of connexins in cancer”), has been demonstrated to prevent temozolomide resistance in human glioblastoma cell lines [65]. The use of peptides arguably provides for a more specific targeting compared with the more general chemical GJIC inhibitors and may reduce potential adverse effects. Cx43-blocking antibodies also reduce tumour growth in murine models. Notably, the intravenous administration of a monoclonal antibody targeting the second extracellular loop of Cx43 reduces glioma growth and survival of experimental animals when used alone [88] or in combination with standard cancer therapy approaches [89]. The same antibody has been used as a guidance system to deliver diagnostic markers or therapeutic compounds, such as cisplatin, to Cx43-positive high-grade gliomas [90, 91].

### Targeting of hemichannels

Connexin hemichannels are normally closed but, when activated, they can release autocrine and paracrine signals, such as NAD^+^, glutamate or ATP, that can affect cell proliferation and survival [92]. For instance, ATP release activates the AKT/AMPK/mTOR signalling pathway, thus linking hemichannel activity with cell proliferation and survival [93]. Several lines of evidence suggest that Cx43 hemichannels are involved in promoting tumour growth. Antibodies against the second extracellular loop domain of Cx43, which blocks hemichannels [94], reduce glioma tumours generated with C6 cells in rats [89]. Similarly, the peptide αCT1 increases the sensitivity to chemotherapy in glioma cells, possibly through the inhibition of Cx43 hemichannel activity [65]. On the other hand, Cx43 hemichannels in osteoblast bone cells may provide an intrinsic self-defence mechanism against breast cancer metastasis [95]. Among the other molecules released by Cx43 hemichannels in osteoblasts, ATP acts as a paracrine signal that triggers an inflammatory cascade to inhibit the migration, invasion and anchorage-independent growth of breast cancer cells [96]. Consequently, the antibody blockade of osteocyte Cx43 hemichannels increases bone metastasis in mice [95]. Collectively, these data suggest that Cx43 hemichannel activity in healthy tissue cells may have a beneficial effect by preventing metastasis, whereas hemichannels in tumour cells may favour their growth.

### Targeting of the connexin interactome

Connexins interact with a wide variety of proteins that, independent of channel activity, can affect cancer cell phenotype, including cell growth, migration and differentiation (Fig. 3; recently reviewed in refs. [97, 98]). Strategies that mimic, promote or disrupt some of these specific interactions could be used in cancer therapy. The fact that Cx43-CT is a disordered region favours the use of mimetic peptides that can restore or interfere with its functions. In tumour cells with low levels of Cx43, the restoration of Cx43 function may reduce cell proliferation. In glioma cells, this occurs via the inhibition of the oncogenic activity of c-Src [99]. The mechanism by which Cx43 reduces c-Src activity resides in a short region of Cx43-CT (266–283) that acts as a docking platform for c-Src together with its endogenous inhibitors PTEN and CSK [43]. Notably, the mimetic peptides of this region fused to the TAT-penetrating sequences (TAT-Cx43_266-283_) can mimic the anti-oncogenic effect of Cx43 in glioma cells such as primary GSCs [55, 100].

In contrast, some proteins may favour cell growth or migration upon interaction with Cx43-CT. For instance, Cx43 may interact with p21-activated protein kinase 1, which activates the MAPK p38 to increase the migration of HeLa cells [101], or may compete with tubulin–Smad2/3 interaction causing Smad2/3 release [102] (Fig. 3). Other Cx43-interaction partners regulate the Cx43 protein level or subcellular localisation. Disruption of these interactions may have therapeutic potential. αCT1 is a Cx43-CT mimetic peptide that comprises the ZO-1 PDZ-binding domain of Cx43 fused to a cell-penetration sequence, which was designed as a tool to disrupt the interaction between endogenous ZO-1 and Cx43 [103]. Consequently, αCT1 can increase the size of Cx43 gap junction plaques and increase GJIC to the detriment of hemichannel activity [104]. αCT1 increases the sensitivity of glioma cells to temozolomide [65] and of breast cancer cells to tamoxifen and lapatinib [105]. In an ongoing clinical trial in dogs with naturally occurring high-grade glioma, a slow controlled release of αCT1 encapsulated in a polymer is being tested in combination with chemotherapy [106].

### Enhanced and synergistic therapeutic effects of connexin targeting

#### The bystander effect: kiss-of-life or kiss-of-death?

The bystander effect underpins a key therapeutic strategy in anticancer approaches and significantly enhances suicide gene therapy strategies [107]. A well-established suicide gene therapy approach involves the viral transduction of the herpes simplex virus (HSV) thymidine kinase (TK) gene into cancer cells [108]. The viral TK enzyme phosphorylates the nucleoside analogue ganciclovir (GCV), which causes chain termination during DNA replication and leads to tumour cell death. Many reports in the 1990s revealed that neighbouring tumour cells that were not transduced with the TK/GCV suicide system also died. This was demonstrated in vitro and in vivo to be due to the GJIC-mediated transfer of activated GCV and/or other toxic metabolites [109, 110]. This led to the hypothesis that the induction of connexin expression or re-establishment of GJIC might potentiate the bystander effect [111], a claim substantiated early on, both in vitro [109, 112] and in vivo [113]. This theory is supported by many studies (e.g. refs. [114–117]), including a recent one in breast cancer [118] and liver cancer, in which the co-expression of Cx43 and SUMO1 in liver CSCs increased GJIC and their sensitivity to HSV-TK/GCV therapy in vitro and in vivo [57]. However, other studies reported no evidence for a GJIC-mediated bystander effect [119]. Moreover, GJIC can protect transduced cells from the toxic active GCV by allowing effective drug dilution throughout the cell, preventing cell death in a so-called “Good Samaritan” effect [120, 121].

The bystander effect is important in a wide range of circumstances other than suicide gene therapy. Notably, some chemotherapeutic drugs or radiation-induced metabolites such as DNA damage-induced sensing/signalling molecules or cytoplasmic irradiation responders (reactive oxygen species, reactive nitrogen species, Ca^2+^ and cytokines) can also transmit through GJIC, inducing a bystander effect [120]. However, the strength and type of the signal may have distinct and opposing effects: (1) toxic signals kill neighbouring cells (which may be tumour cells or healthy cells) or (2) toxic signals are diluted into neighbouring cells, favouring the survival of the targeted cells. Importantly, this implicates GJIC in coordinating cellular and tissue responses to carcinogens, radiation and chemotherapies and leads to the possible strategies (via both enhanced or blocked GJIC) for either protecting undamaged non-targeted cells against such signals or potentiating the signals in order to kill tumour cells. Application of this knowledge to other aspects of connexin cancer biology is important.

#### Need for combinatorial therapy

Within the field of cancer therapy, there is a general consensus on the need for rational combinatorial targeted therapy. Synergistic effects on cancer cell growth as a result of enhanced connexin expression or GJIC in combination with drugs targeting other cellular processes have been described in many studies [122–127] (Table 3). For instance, kanglaite, a natural plant seed compound that upregulates Cx43 expression, sensitises colorectal cancer cells to Taxol [124]. Simvastatin (a statin) induces GJIC and enhances the effect of platinum-based chemotherapeutic drugs [126]. In addition, protection can be provided against the non-desired cytotoxic effects of cisplatin on healthy cells, including reproductive testicular Sertoli cells [127]. Such dual kiss-of-life/kiss-of-death bystander effects can contribute to the overall chemotherapeutic outcome on multiple levels. It also poses a therapeutic challenge. The putative positive and negative effects such therapies can provide in relation to tumour growth must therefore be carefully assessed in order to avoid a possible worsening rather than an improvement of the clinical outcome.

The therapeutic response of cancer cells can also be affected by the direct overexpression of connexins, in an isoform-specific manner. For instance, Cx43 overexpression can enhance the sensitivity to common chemotherapeutic drugs such as doxorubicin, fluorouracil and oxaliplatin in human gastric cancer cells [128], etoposide, paclitaxel and doxorubicin in glioma cells [129], and artesunate in MCF-7 breast cancer cells [130]. Cx32 potentiates the cytotoxicity of vinblastine and Src inhibitors in renal cell carcinoma cells [131], whereas Cx26 increases the effect of cisplatin in human bladder cancer cells [132] and of doxorubicin in prostate cancer cells [133]. Both channel-dependent and -independent mechanisms have been suggested to underlie this effect [130, 134].

In other contexts, connexins can have an inverse effect with respect to drug sensitivity. Reduced Cx43 expression is associated with increased drug sensitivity in glioma cells [52, 134–136], whereas the upregulation of Cx26 is associated with gefitinib resistance in lung cancer cells [137]. The specific inhibition of Cx46-mediated GJIC in GSCs attenuates proliferation, self-renewal and tumour growth and synergises with temozolomide to induce apoptosis [138]. There is also in vivo evidence to support this dichotomy in relation to therapeutic efficacy. For instance, shRNA-mediated knockdown of Cx43 or inhibition of GJIC by meclofenamate and tonabersat strongly potentiates the effect of carboplatin-based chemotherapy on brain metastases from breast and lung carcinoma cells [50]. Moreover, the efficacy of tumour necrosis factor-related apoptosis-inducing ligand therapy is enhanced when combined with the GJIC inhibitor carbenoxolone in an intracranial glioma model [136]. The combined use of tumour necrosis factor-related apoptosis-inducing ligand and carbenoxolone could offer a favourable alternative for the treatment of glioma, particularly considering the low cytotoxic nature of carbenoxolone. Notably, carbenoxolone, as well as peptides targeting Cx43, attenuates cancer-induced bone pain [139], highlighting another facet of cancer care in which connexins can be further explored.

## Emerging concepts

### Role of connexins in tumour microenvironment

**Fig. 4:**
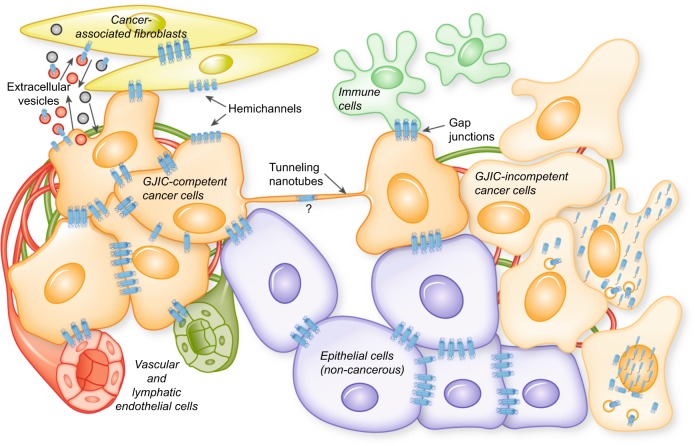
Connexins and the tumour stroma. GJIC can occur between cancer cells or in a heterocellular manner between cancer cells and nearby cells such as noncancerous epithelial tissue cells and stromal cells, including cancer-associated fibroblasts, immune cells and vascular and lymphatic endothelial cells. In addition, there is crosstalk via the hemichannel release of autocrine and paracrine signals. These signals influence tumour growth both positively and negatively in a context-dependent manner and help to regulate apoptosis, proliferation, invasion, intravasation and extravasation. In addition, connexins are thought to be implicated in other communication forms, as a part of tunnelling nanotubes (microtubes) or extracellular vesicle function (e.g. exosomes). Other tumours, or parts of tumours, are devoid of GJIC and may or may not express connexins at high levels in the cytoplasm or nucleus, thus escaping the direct GJIC with surrounding cells. This may be associated with a reduced polarity and cell–cell adhesion. The benefits and drawbacks of maintained GJIC are likely tissue and stage dependent. An understanding of this complex network of signals is essential to move forward with additional therapeutic strategies of targeting connexins in cancer. GJIC, gap junction intercellular communication

That GJIC between normal cells and tumour cells (heterologous GJIC) can inhibit the growth of tumour cells was shown in the mid 1980s by Loewenstein and colleagues [140]. This phenomenon has since been demonstrated in many cancer types and model systems, as recently reviewed [141]. Heterologous GJIC between tumour cells and the cells within their microenvironment (Fig. 4) has been linked to both positive and negative effects on tumour progression and therapy resistance.

#### Communication between cancer cells and immune cells

Tumour cells deliver antigenic peptides through gap junctions to dendritic cells, and this cross-presentation is associated with enhanced immune-mediated tumour elimination. Presentation of antigenic peptides between melanoma cells and dendritic cells increases the melanoma-specific T-cell response [142]. The infection of melanoma tumours with *Salmonella* bacteria induces Cx43 expression, which enhances the antigen presentation into infiltrating dendritic cells, activating an antitumour response [143]. Cx43-mediated GJIC may also improve the immune surveillance of tumours via the activation of natural killer cells [144, 145]. GJIC-mediated transfer of miRNAs from immune cells to hepatocarcinoma cells seems to inhibit tumour growth [146]. These studies (and others, as reviewed recently [141]) are of particular interest in relation to immunotherapy and tumour vaccines [141]. Connexins also regulate many other functions within the immune and lymphatic systems, which awaits an additional analysis [147].

#### Angiogenesis and communication between cancer cells and endothelial cells

Angiogenesis, the formation of new capillaries from pre-existing blood vessels, is required for cancer progression [148]. Overexpression of Cx43 in melanoma and breast cancer cells suppresses tumour angiogenesis [28, 149]. In accordance with these findings, Cx43 knockdown in melanoma cells increases vessel density [149]. The silencing of Cx43 in breast cancer cells results in increased vascular endothelial growth factor expression and decreased thrombospondin expression [29]. In contrast, the overexpression of Cx26 or Cx43 in breast cancer cells is associated with the upregulation and secretion of IL-6 and MCP-1, which inhibit endothelial cell tube formation in vitro and tumour vascularisation in vivo [28].

Endothelial connexins also play important roles in tumour angiogenesis. The knockdown of endothelial Cx37, Cx40 or Cx43 or the pharmacologic inhibition of GJIC diminishes the angiogenic sprouting of endothelial cells in in vitro studies [150]. A recent work using a combination of different in vitro, ex vivo and in vivo models has shown that targeting of endothelial Cx40 decreases tumour growth by reducing angiogenesis and improving vessel perfusion [87]. This was demonstrated both in mouse endothelial-specific Cx40 knockout models and by the injection of the specific peptide ^40^Gap27 that binds to the extracellular loop of Cx40 and blocks channel activity [151].

Because endothelial and myoendothelial gap junctions are fundamentally important for a coordinated vessel response over longer distances [152, 153], a therapeutic approach focused on vascular connexins would be conceivable through the specialised targeting of tumour vessels in combination with anti-angiogenic strategies.

There is also evidence of direct communication between tumour cells and endothelial cells (Fig. 4). Recent studies have demonstrated GJIC-mediated heterotypic exchange of microRNAs between cancer cells and endothelial cells. Notably, endothelial cells deliver miR-145–5p to colon cancer cells via gap junctions, causing the upregulation of Cx43 expression and reduced angiogenesis [154]. Moreover, the inhibition of GJIC by carbenoxolone blocks the interchange of specific cancer-associated microRNAs between human microvascular endothelial cells and a human glioma cell line [155].

As mentioned, Cx26 may facilitate extravasation into the endothelium during metastasis [47], and Cx43 enhances attachment and diapedesis into endothelial cells in models of breast cancer [48, 156] and melanoma metastasis [157]. Heterocellular Cx43-mediated GJIC between gastric cancer cells and mesothelial cells may facilitate diapedesis during peritoneal metastasis [75]. Using zebrafish and chicken embryo models of brain metastasis, Stoletov et al. [158] determined that Cx26 and Cx43 acted in the early initiation stages of metastatic lesion formation in association with vasculature. RNAi-mediated depletion of Cx26 and Cx43 in melanoma and breast cancer cells, respectively, or pharmacological inhibition of GJIC using carbenoxolone, was found to inhibit brain colonisation by blocking tumour cell extravasation and blood vessel co-option [158]. Such crosstalk between cancer cells and blood vessels may also occur via hemichannels, such as the one through ATP release, which ultimately can stimulate angiogenesis [159]. There is also evidence that GJIC facilitates cancer cell migration through the lymphatic endothelium [160]. Thus, blockade of connexin-mediated heterocellular communication is emerging as a potential viable strategy for reducing metastasis. Indeed, the Cx43 channel blocker oleamide has antimetastatic properties in the MDA-MB-231 breast cancer cell line, presumably due to the inhibition of extravasation into the endothelium [85].

#### Communication between cancer cells and astrocytes

Connexins can also elicit pro-tumourigenic effects in brain tumours. Cx43 expression and heterologous GJIC between malignant glioma cells and reactive astrocytes enhance cell invasion into the brain parenchyma [161–163], which may in part occur through microRNA-mediated signalling [164]. Glioma cells can also become more resistant to chemotherapy- or radiotherapy-induced cell death through both homocellular and heterocellular GJIC pathways [51, 53, 165], as well as via Cx43-specific GJIC-independent mechanisms [166]. GJIC between reactive astrocytes and melanoma cells protects against chemotherapy through the sequestration of calcium [34]. A recent study has described another signalling network between astrocytes and breast and lung carcinoma cells involving cGAMP and the stimulator of interferon genes (STING) pathway (see section “Modulation of connexin expression and GJIC”), and the inhibition of GJIC or Cx43 expression was found to substantially reduce the brain metastatic burden and enhance chemotherapy efficacy [50]. In summary, GJIC between tumour cells and astrocytes promotes colonisation, resistance to chemotherapy and survival of tumour cells in the brain [34, 50, 164, 167]. Targeting of this axis may provide clinical benefits.

### Connexins and tumour viruses

An estimated 10–12% of human cancers worldwide are caused by viruses and 4.5% are caused by the so-called “high-risk” human papillomaviruses (HPVs) [168]. Rous sarcoma virus-infected fibroblasts display a loss of GJIC, and its oncogene pp60^v-src^ causes Cx43 tyrosine phosphorylation and reduced Cx43 levels [169]. Similarly, SV40 large T antigen-transformed normal invasive trophoblasts lose GJIC and display reduced Cx43 levels [169]. Human cytomegalovirus is not recognised as a tumour virus. However, human cytomegalovirus proteins have been detected at high levels in gliomas and can downregulate Cx43 and GJIC [170]. These data suggest that a range of viruses inhibit GJIC.

In 1969, McNutt and Weinstein recognised that gap junction plaques are lost in cervical cancers [171]. Subsequently, cervical cancers were shown to be caused by HPVs [172]. Reduced connexin expression can occur in preneoplastic cervical lesions [173], perhaps in part due to the loss of epithelial differentiation. On the other hand, loss of Cx43-mediated GJIC occurs as a direct consequence of HPV-associated cancer progression [174], and HPV infections alter multiple connexins at the transcript level [175]. High-risk HPVs encode two oncoproteins, E6 and E7, which are highly expressed in cervical cancer. E6 alters Cx43 trafficking to the plasma membrane either through its ability to alter cell signalling pathways or through its interaction with the Cx43 partner protein Dlgh1 [42, 176]. HPV E5, a subsidiary oncoprotein, also downregulates Cx43 and GJIC when overexpressed in keratinocytes [177].

Upon infection, viral DNA is detected by cyclic GMP-AMP synthase, which synthesises the STING activator cGAMP to induce an antiviral state through intercellular transmission. Connexins are responsible for the bystander immunity to viruses [178], and so viruses may have evolved means to inactivate this response by regulating connexins, a feature that could be exploited therapeutically.

### Role of connexins in long-distance cell–cell communication

#### Tunnelling nanotubes

Tunnelling nanotubes (TNTs) are thin actin-based membrane bridges that connect cells over distances of up to several cell diameters [179]. These structures allow for the intercellular transfer of microRNAs, proteins and cytoplasmic organelles, including mitochondria. The presence of connexin channels interposed in the nanotube connections permits long-distance electrical coupling between cells [179, 180]. TNTs are formed between various cancer cell types in vitro [179]. TNTs between malignant cells and stromal cells may be involved in chemoresistance [181] and tumour–stromal crosstalk [182, 183]. Recently, Osswald et al. [184] demonstrated in vivo that glioma cells form a network of TNT-like structures called microtubes that contribute to their invasion in the brain. Cx43-containing gap junctions within this network were suggested to turn the tumour into a syncytium of interconnected cells that is highly resistant to radiation therapy, presumably by distributing calcium between cells to prevent apoptosis upon radiation-induced release of intracellular calcium [184].

#### Extracellular vesicles

Extracellular vesicles (EVs), which constitute microvesicles, apoptotic bodies and exosomes, are membrane-based structures that can carry and deliver bioactive molecules, including proteins and nucleic acids, from one cell to another. This form of cell-to-cell communication can occur over very long distances and efficiently cross the blood–brain barrier. EVs released by tumour cells and cancer-associated fibroblasts affect cancer progression by transferring molecules influencing tumour initiation, angiogenesis, metastasis and drug resistance [185]. Functional Cx43 channels were recently identified in the membrane of EVs [186]. In melanoma, Cx32, Cx43 and Cx45 were detected in these structures [187]. Connexins facilitate the transfer or exchange of EV contents with target cells, possibly by improving fusion events with cells [186]. This may be exploited therapeutically to improve drug delivery. However, in one mouse model, delivery of doxorubicin as a chemotherapeutic agent was not improved in the presence of Cx43 in EVs, although it strikingly reduced cardiotoxicity [188]. In addition, Cx43 phosphorylation through extracellular signal-regulated kinase signalling induces exosome release upon traumatic brain injury [189]. Thus, it seems clear that connexins are involved in the formation and function of EVs, but the therapeutic implications await further works.

## Concluding remarks and future perspectives

Substantial knowledge on how gap junctions contribute to cancer has accumulated since the seminal work by Loewenstein and Kanno demonstrated a loss of electrical coupling in liver cancer more than 50 years ago [7]. Connexins predict the prognosis of a number of cancers, although the lack of established protocols and confirmatory independent studies has limited their clinical utility. Several promising studies using an expanded set of tools to modulate connexin or gap junction function have demonstrated potent antitumoral effects. As we decipher their cancer type- and stage-specific roles, significant progress towards targeting of connexins and gap junctions in a patient-specific therapeutic setting can be expected. An ongoing clinical trial testing the GJIC inhibitor meclofenamate in patients with carcinoma metastasis to the brain underpins this positive outlook (https://clinicaltrials.gov/ct2/show/NCT02429570?id=NCT02429570&rank=1&load=cart). Nevertheless, there are significant challenges that remain to be addressed. The fact that connexins can be both anti- and pro-tumorigenic is of particular concern. For example, would enhancing GJIC or connexin expression in a primary tumour run a risk of more efficient metastatic spread or growth? Does inhibition of GJIC in patients with metastasis increase the risk of further tumour dissemination or re-activated tumour growth in sites harbouring dormant tumour cells? Currently, this clinical problem can only be addressed by hypothetical risk assessment and thus further research, including an extensive use of in vivo models and a careful follow-up of ongoing clinical trials, is required.

Currently, only a few of the 21 connexin isoforms have been characterised in terms of their role in cancer. Additional studies are necessary to elucidate the GJIC-dependent and -independent mechanisms by which the various connexins positively or negatively affect cell growth, differentiation, invasion and other important cancer-associated features. In this context, more specific tools will be required to target the different functions of connexins. Further efforts need to be devoted to the identification of more specific connexin inhibitors. The use of specific peptides and peptidomimetics have shown a great promise towards this. However, their side effects must be carefully addressed (particularly if applied systemically) due to the critical functions of several connexins in various organs. Another important challenge will be to dissect the molecular basis of the regulation of the various connexin isoforms at the transcriptional, translational and post-translational levels and to define how the dysregulation of these processes contributes to aberrant levels or subcellular localisation of connexins during various stages of cancer progression. The identification of solutions to these research challenges will set the stage for new diagnostic and therapeutic advances.
